# 299. D-dimer as an ICU Admission Risk Predictor in COVID-19 Patients, A Prospective Study

**DOI:** 10.1093/ofid/ofab466.501

**Published:** 2021-12-04

**Authors:** Oriana Narváez - Ramírez, Lina Morales-Cely, Ingrid G Bustos-Moya, Yuli Viviana Fuentes-Barreiro, Julian Lozada-Arciniegas, Elsa Daniela Ibañez-Prada, Laura A Bravo-Castelo, Daniela Parra-Tanoux, Paula Ramirez, Salome Gomez-Duque, Enrique Gamboa-Silva, Edar Caceres, Luis F Reyes

**Affiliations:** 1 Universidad de la Sabana, Bogota, Distrito Capital de Bogota, Colombia; 2 Universidad de la Sabana, Chía, Colombia, Bogota, Cundinamarca, Colombia; 3 Universidad de La Sabana, Bogota, Distrito Capital de Bogota, Colombia

## Abstract

**Background:**

Since the onset of the 2019 coronavirus disease 2019 (COVID-19) pandemic, the rapid increase in community-acquired pneumonia (CAP) cases has led to an excessive rate of intensive care units (ICU) admissions, a rate varying between 5-18%, depending on the country. Consequently, the study of serum biomarkers, such as D-dimer, have been utilized to identify patient with severe disease. However, further data is needed to confirm the association between this serum concentration of D-dimer and the risk of ICU admission. Thus, the aim of this study was to determine if serum concentration of D-dimer predict the risk of ICU admission in patients with COVID-19 and CAP.

**Methods:**

A prospective observational study was carried out at the Clinica Universidad de La Sabana, Colombia. Patients older than 18 years old, hospitalized for COVID-19 or CAP were included. Then, patients were stratified into ICU and non-ICU patients. Plasma samples were collected within the first 24 hours of hospital admission to quantify D-dimer using the PATHFAST system. Concentrations were compared among groups and to assess the biomarker capacity to predict ICU admission risk, ROC curves were used. Finally, a DeLong test was applied to compare their differences.

**Results:**

A total of 240 patients diagnosed with lower respiratory tract infection were included in the study. 88 patients were COVID-19 negative (CAP) and 152 were positive. Plasma concentrations of D-dimer (µg/ml) were significantly higher in COVID-19 patients admitted to the ICU when compared with non-ICU COVID-19 admitted patients (Median [IQR]; 1.54 [0.9-3.25] Vs. 1.13 [0.69-1.69], p=0.005). The area under curve (AUC) ROC to predict ICU admission was 0.62 among COVID-19 patients. DeLong’s test p value was 0.24.

Serum D-dimer an ICU admission

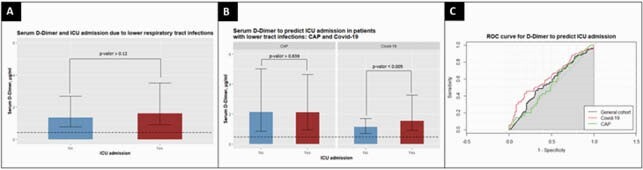

**Conclusion:**

D-dimer seems to be a promising tool to identify COVID-19 patients with disease. However, this predicting capacity was not observed in CAP patients. Further studies are needed to identify the mechanisms underling the elevation of D-dimer in COVID-19 patients.

**Disclosures:**

**All Authors**: No reported disclosures

